# The effectiveness of individual interpersonal psychotherapy as a treatment for major depressive disorder in adult outpatients: a systematic review

**DOI:** 10.1186/1471-244X-13-22

**Published:** 2013-01-11

**Authors:** Madelon L J M van Hees, Thomas Rotter, Tim Ellermann, Silvia M A A Evers

**Affiliations:** 1 Caphri, School of Public Health and Primary Care; Faculty of Health, Medicine, and Life Sciences, Maastricht University, Maastricht, the Netherlands; 2 College of Pharmacy and Nutrition, University of Saskatchewan, Saskoon, Canada; 3 Institute for Public Health and Nursing Research (IPP), University of Bremen, Bremen, Germany; 4 Caphri, School of Public Health and Primary Care; Department of Health Services Research, Maastricht University, Maastricht, the Netherlands

**Keywords:** Interpersonal psychotherapy, Major depressive disorder, Systematic review

## Abstract

**Background:**

This systematic review describes a comparison between several standard treatments for major depressive disorder (MDD) in adult outpatients, with a focus on interpersonal psychotherapy (IPT).

**Methods:**

Systematic searches of PubMed and PsycINFO studies between January 1970 and August 2012 were performed to identify (C-)RCTs, in which MDD was a primary diagnosis in adult outpatients receiving individual IPT as a monotherapy compared to other forms of psychotherapy and/or pharmacotherapy.

**Results:**

1233 patients were included in eight eligible studies, out of which 854 completed treatment in outpatient facilities. IPT combined with nefazodone improved depressive symptoms significantly better than sole nefazodone, while undefined pharmacotherapy combined with clinical management improved symptoms better than sole IPT. IPT or imipramine hydrochloride with clinical management showed a better outcome than placebo with clinical management. Depressive symptoms were reduced more in CBASP (cognitive behavioral analysis system of psychotherapy) patients in comparison with IPT patients, while IPT reduced symptoms better than usual care and wait list condition.

**Conclusions:**

The differences between treatment effects are very small and often they are not significant. Psychotherapeutic treatments such as IPT and CBT, and/or pharmacotherapy are recommended as first-line treatments for depressed adult outpatients, without favoring one of them, although the individual preferences of patients should be taken into consideration in choosing a treatment.

## Background

Major depressive disorder (MDD) is a mental disorder characterized by a depressed mood, diminished interest or pleasure, sleeping problems and tiredness, and negative thoughts [1]. The mean one-year-prevalence of depression in European inhabitants between 18 and 65 years old is 6.9% [2], and 16.2-16.6% of US adults develop a major depressive disorder [3,4]. Furthermore, depression causes a high burden worldwide, taking fourth place in a ranking of leading contributors to the burden of diseases in 2000. In 2020, it is estimated that depression will take second place in the ranking for all ages and sexes [5]. Moreover, depression is the leading cause of years of life lived with disability, in all ages and sexes, accounting for 11.9% of all disability [6]. Since it appears that persons suffering from mental disorders make more use of health care services [7], the increasing prevalence of depression leads to an increase in health care costs.

Research [8] and Dutch guidelines [9] suggest treating depression with psychotherapy and/or medication. Psychotherapy follows several kinds of methodologies. For depression, Cognitive (Behavior) Therapy (CBT) and Interpersonal Psychotherapy (IPT) are often applied. CBT originates from behavior therapy and cognitive therapy, and combines elements of both therapies [10-12]. IPT was originally developed for treating acute depression by improving the interpersonal functioning with important others [13-17]. This study will focus on the effectiveness and efficacy of IPT, since CBT has been subject of many studies up until now, while IPT has only recently become a subject of interest.

As a monotherapy for adults, individual IPT appears to be an effective treatment for depression [18-20], and several reviews [21-25], and meta-analyses [26-33] have been performed on the effectiveness of all kinds of methodologies of psychotherapy. Nevertheless, psychotherapy is a broad concept, and reviews and meta-analyses have often focused on different combinations of psychotherapy for treating depression without comparing one specific sole treatment to another [21,25-30,32,34]. Furthermore, although sole individual IPT appears to be effective, few reviews focus on sole individual IPT in adults with MDD as a primary diagnosis. Often, dissimilar study populations are compared with each other, for example adult, adolescent, and elderly patients in one study [23,25-30,33-35]. Furthermore, several more types of depression exist: dysthymic disorder or depression with medical conditions, for example, but this review will focus only on MDD. Chronic MDD and postpartum depression (PPD) will be included in this systematic review, for the following reasons. First of all, treatment for patients with chronic and non-chronic depression is equal in terms of content and structure. Therefore, the treatments of these patient groups are comparable. Secondly, the symptoms of both kinds of depression are comparable in terms of severity and content, which makes the patients comparable. Furthermore, women with PPD experience the same kind of symptoms as patients with MDD.

Since comorbidity is very common in patients suffering from depression, and this possibly increases the severity of the depression [36-44], this review will focus on MDD as a primary diagnosis with possible comorbidity.

Other factors influencing the results of previously executed systematic reviews include different age groups, in which form the provided IPT is administered, distinct settings, and the time periods during which the studies were executed. IPT is often adjusted for applicability to elderly [45] or adolescent [46] depressed patients, or in the form of group IPT [47]. Therefore, these kinds of treatments may be hard to compare with each other. That is why this review focuses on individual IPT. From here on, when IPT is described in the review, individual IPT is meant, unless described otherwise. Furthermore, the setting in which treatment takes place suggests the depression’s level of severity. It is assumed that inpatients have a more severe depression, which is harder to treat. In addition, inpatient care is often multidisciplinary, which makes it difficult to examine the effects of separate therapies. Research has been conducted on IPT since the 70s, which is why the date limit for this review is set on 1970. This review will give an overview of studies published between January 1970 and August 2012, with a focus on sole IPT administered to adults. Since some therapies have an effect relatively quickly, we did not apply a minimum for duration of a therapy.

With all of the above in mind, the aim of this study is to give an overview of recent literature describing the effectiveness and efficacy of sole individual IPT in comparison with standardized forms of treatment for treating patients with MDD as a primary diagnosis. The following research question has been formulated: *Is individual interpersonal psychotherapy more preferable in comparison with other standardized forms of treatment for treating adult outpatients with a primary diagnosis of major depressive disorder?*

In order to answer this question, a systematic review will be performed on RCTs and C-RCTs comparing the effectiveness (the outcome of a new treatment compared to other kinds of treatment(s), usually in a clinical setting) or efficacy (the outcome of treatments in homogeneous patient groups, usually in an experimental setting) [48] of individual sole IPT with other standardized forms of treatment, for treating adult outpatients with MDD as a primary diagnosis.

## Methods

This paragraph will outline which steps were taken in order to perform this systematic review. An overview of the methods used for data collection, study selection, and data analysis will be provided.

### Data sources

RCTs about IPT for depression were collected by searching PubMed and PsycINFO for studies published between January 1970 and August 2012. The following medical subject heading (MeSH) categories and keywords were used: depression, postpartum depression, major depressive disorder, dysthymic disorder, interpersonal psychotherapy, treatment outcome, clinical trials. The exact search terms and MeSH headings can be found in the additional files (Additional file 1 – Search strategy). All titles and abstracts were screened, and only studies which met the review inclusion criteria (see next paragraph and Table 1) were selected for further review. Citation tracking and snowballing techniques added studies to the second screening phase, in which selected studies were screened for eligibility using a predefined checklist (see Data analysis) (Additional file 2 – Checklist).

**Table 1:** Summary of inclusion and exclusion criteria

***Study characteristic***	***Inclusion criteria***	***Exclusion criteria***
*Type of study*	Randomized controlled trial	
	>1970	<1970
	English language	Other languages
*Population*	Adults (18–65)	Elderly people or adolescents
	Major depressive disorder as a primary diagnosis	Bipolar disorder
*Interventions*	Individual sole IPT	Group IPT
		IPT combined with other therapy
*Comparators*	Other evidence-based psychotherapies, combined treatment, or pharmacotherapy	Alternative therapy, bibliotherapy, complementary therapy, counseling, psychoeducation, supportive therapy
*Setting*	Outpatient ambulant care, primary care	Inpatient care
	Western jurisdictions	Outside of Western jurisdictions
*Outcome*	Depressive symptoms	

*IPT* Interpersonal Psychotherapy.

### Study selection

Only studies with sufficient methodological quality meeting the inclusion criteria were selected for this review. The criteria for selection will be described shortly. An overview of the inclusion and exclusion criteria is provided in Table 1.

Studies were included if they were randomized or cluster-randomized evaluations (RCTs or C-RCTs) published in English after January 1^st^, 1970, and took place in western jurisdictions, to ensure high internal validity. These studies had to focus on MDD (non-chronic or chronic) as a primary diagnosis in adults (18–65 years old). The diagnosis must have been reached using a formal classification system, such as the Diagnostic and Statistical Manual of Mental Disorders (DSM) [1], the International Classification of Diseases (ICD) [49], or the Research Diagnostic Criteria [50]. Bipolar disorders as primary diagnoses were excluded, as well as cases where the patients were elderly people or adolescents, or in cases in which physical conditions might contribute to the (severity of) depressive symptoms. The proposed intervention must have been individual sole IPT, in comparison with other psychotherapies, pharmacotherapy, or combined treatment. Group IPT and other kinds of treatments were excluded. Studies executed in ambulant care or primary care were included, whereas inpatient care patients were excluded.

By making the inclusion criteria very strict, a more homogeneous group, with a narrower scope, was created, which made it possible to focus on clinical applicability of the treatments for these kinds of patients.

### Data analysis

Before the data were analyzed for this review, the methodological quality of the studies included after screening has been assessed, using a predefined checklist (Additional file 2 – Checklist). This checklist was composed of Delphi-list questions [51] and questions assessing the risk of bias in effect evaluation studies [52]. General questions were composed for collecting relevant information about the study, after which the resulting information was entered in a Microsoft Excel table for a clear overview. This overview was used to create a table of evidence of the extracted study data, and to summarize the most important findings. MH performed the analysis and consulted TR in case of doubt. In this case, the analyses were double checked and consensus was reached.

## Results

**Figure 1:**
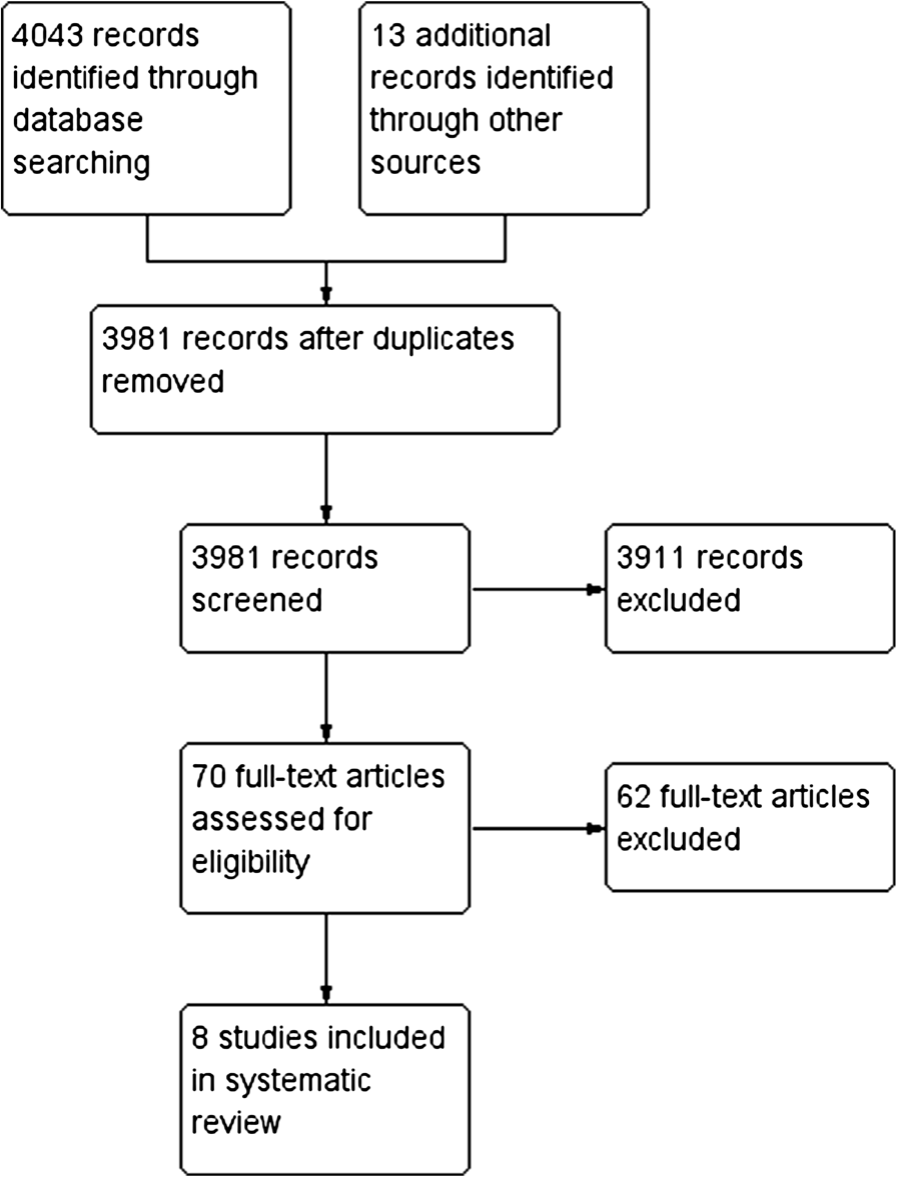
**Flow diagram of included and excluded articles; reasons in Additional file**3**.**

The literature search resulted in 3981 studies, of which 3911 were excluded from further review for several reasons, documented below. Figure 1 shows the flow diagram of included and excluded studies. Studies were excluded when they did not meet the inclusion criteria, based on the title and abstract: i.e. they did not focus on MDD as a primary diagnosis, on individual sole IPT, or the target group was anything other than adults. Another 62 were excluded after reading the full text, leaving 8 articles eligible for this review.

These 62 full-text articles were excluded for the following reasons: being reviews or meta-analyses [21-23,25-30,32,34,53-60], being a protocol for a study [61], being a study based on earlier/other studies [43,45,62-76], there was no comparison in the study [77], MDD was not the primary diagnosis [78-82], the study had the wrong aim for this review [83-86], there was no research data [87-91], or one of the interventions was not IPT as described in the eligibility criteria [35,92-100]. See Additional file 3 – List of excluded studies for a detailed description of the reasons for exclusion.

### Description of the studies

The main characteristics of the RCT studies included are summarized in Table 2. One study was carried out in the Netherlands [101], one in New Zealand [102], one in Canada [103], one in the UK [104], one in Germany [105], and three in the USA [106-108]. All studies clearly described eligibility criteria and success-of-treatment point. All but two [103,104] included an intention-to-treat analysis. Seven studies reported comparable sociodemographic and psychiatric variables at baseline. One [103] did not report these variables.

**Table 2:** Summary of the characteristics of the includes studies

**Study**	**N included**	**N completed treatment**	**Population and primary diagnosis**	**Treatment types**	**Duration (weeks)**	**Primary and secondary outcome measure**	**Time between pre- and post-treatment**
*Blom et al. (2007)*	193	132	Adults with MDD	IPT vs.Nefazodone vs.IPT+nefazodone vs.IPT+placebo	16	HAMD	12 weeks
						MADRS	
*Elkin et al. (1989)*	250	155	Adults with MDD	IPT vs. CBT vs. IMI-CM vs. PLA-CM	16	HRSD	16 weeks
						BDI	
*Luty et al. (2009)*	177	159	Adults with MDD	IPT vs.CBT	16	MADRS	16 weeks
						HRSD	
						BDI	
*Marshall et al. (2008)*	159	102	Adults with MDD	IPT vs.CBT vs.PHT-CM	16	HRSD	16 weeks
*Martin et al. (2001)*	28	28	Adults with MDD	IPT vs.Venlafaxine	16	HAMD	6 weeks
						BDI	
*O’Hara et al. (2000)*	120	99	Women with PPD	IPT vs. WLC	12	HRSD	12 weeks
						BDI	
*Schramm et al. (2011)*	30	29	Adults with early onset chronic MDD	IPT vs.CBASP	16 with 12 months follow-up	HRSD	16 weeks
						BDI	
*Schulberg et al. (1996)*	276	150	Adults with MDD	IPT vs.Nortriptyline vs.Usual care	16 with 8 months follow-up	HRSD	8 months

*CBASP* Cognitive Behavioral Analysis System of Psychotherapy; *CBT* Cognitive Behavior Therapy; *HAMD* Hamilton Depression Rating Scale; *HRSD* Hamilton Rating Scale Depression; *IMI-CM* imipramine plus clinical management; *IPT* Interpersonal Psychotherapy; *MADRS* Montgomery-Åsberg Depression Rating Scale; *MDD* Major Depressive Disorder; *PHT-CM* pharmacotherapy plus clinical management; *PLA-CM* placebo plus clinical management; *WLC* wait list condition.

A total of 1233 patients were included in the review, of whom 854 completed treatment in outpatient facilities. Of the patients included, 392 received IPT, 14 received CBASP (Cognitive Behavioral Analysis System of Psychotherapy), 160 received CBT, 153 received pharmacotherapy (nefazodone, nortriptyline hydrochloride, or venlafaxine hydrochloride), 67 received pharmacotherapy plus clinical management, 49 received IPT and nefazodone, 47 received IPT and a placebo, 34 received a placebo plus clinical management, 92 received usual care consisting of communication with a physician for appropriate treatment, and 51 were put on a wait list. The mean age in seven studies [101,102,104-106] ranged from 29.4 to 40.2 years old, and the percentage of female patients varied from 55% to 83%, except for one study, in which only females participated [108]. One study did not report these data [103]. All patients were diagnosed with non-psychotic MDD as a primary diagnosis according to the DSM-III-R [109], DSM-IV [110], or the Research Diagnostic Criteria [50].

IPT in all studies was based on a standardized manual [14,17], as was CBASP [111] and CBT [12,112,113]. The number of IPT and CBT sessions varied from 8 to 24 in a 12- or 16-week period, and most of the sessions were held weekly. Physicians administering nefazodone or nortriptyline were instructed to follow a manual. Patients receiving nefazodone started at 100 mg capsules per day, and doses were gradually increased to a minimum of 400 mg, with a maximum of 600 mg [101]. Patients receiving nortriptyline started at 25 mg per day, aiming for blood levels of 190–570 nmol/liter [106]. Patients receiving imipramine hydrochloride had a dosage between 150 and 185 mg. Pharmacotherapists administering venlafaxine followed an evidence-based protocol of 37.5 mg twice-daily doses [104]. Pharmacotherapy plus clinical management was administered by a psychiatrist who followed the client for the duration of the protocol associated with the antidepressant medication [103], or as long as the clinical management would be administered [107].

### Risk of bias

**Figure 2:**
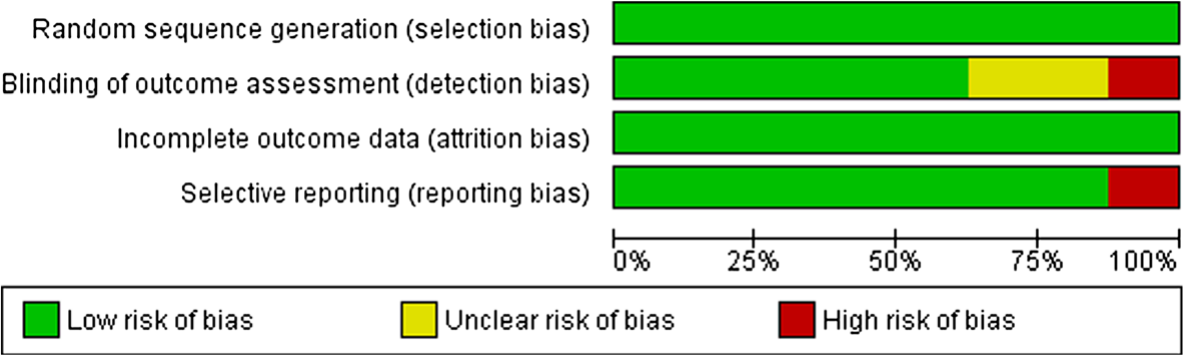
Summary of risk of bias in six studies.

Risk of bias was measured and summarized (see Figure 2) according to the standards of the Cochrane Collaboration [52]. Although this was not always described exhaustively, all studies used randomization and seemed to present complete outcome data. Therefore, all included studies had a low risk of selection bias and attrition bias. Nevertheless, two studies [103,107] had an unclear risk of detection bias and one of them [103] had a high risk of reporting bias. Another study [108] had a high risk of detection bias. Notwithstanding these higher levels of bias, these studies have been included in this review.

### Findings on outcome measurements

The outcome of the HAMD showed an overall decrease in the level of depression over time (*p*<0.001) between the four treatment conditions (IPT, nefazodone, IPT and nefazodone, IPT and placebo), but this was not statistically significant. A significant difference was found between IPT and nefazodone and the use of nefazodone without IPT in favor of the first (for the intent to treat sample: adjusted OR (95% CI)=3.22 (1.02-10.12), *p*=0.045). Furthermore, a significant difference was found in the MADRS scores. Patients receiving IPT with nefazodone improved more than did patients receiving nefazodone without IPT. Furthermore, the nefazodone condition showed only a small improvement after the first six weeks [101].

Imipramine hydrochloride combined with clinical management (CM) was significantly superior to placebo with CM on general level of functioning. Patients receiving IPT or imipramine hydrochloride with CM appeared to have a better outcome on the HRSD than patients receiving placebo with CM (*p*=0.018 and *p*=0.017). Furthermore, these patients showed a significantly higher percentage in the recovery analysis compared to placebo with CM patients, measured by a score of six or lower on the HRSD (*p*=0.010 and *p*=0.013) [107].

In the Luty et al. study [102], depressive symptoms improved for about 55%. No statistically significant differences were found between IPT and CBT on the primary outcome measure MADRS (9.5% mean difference (95% CI), *p*=0.059), nor after controlling for baseline severity (*p*=0.055).

HRSD scores were significantly higher in the IPT condition compared to the PHT-CM condition (*t*(96)=−2.46, *p*<0.05, *d*=−0.50). No significant differences were found between IPT and CBT conditions (*t*(96)=−1.19, *p*=0.46, *d*=−0.24), or between CBT and PHT-CM conditions (*t*(96)=−1.35, *p*=0.37, *d*=−0.28) [103].

Depressive symptoms, measured by the HAMD and BDI, improved significantly (*p*<0.001) in the first six weeks for patients receiving IPT or venlafaxine [104]. Although the venlafaxine group showed a slightly better outcome than the IPT group, no significant differences were found after six weeks.

O’Hara described recovery rates for women with PPD based on HRSD scores and BDI scores, favoring IPT over wait list condition (WLC). Based on HRSD scores (HRSD ≤6), IPT had a recovery rate of 31.7%, compared to 15% of WLC (*p*=0.03). Based on BDI scores (BDI ≤9), IPT had a recovery rate of 38.3%, while women in the WLC group showed a recovery rate of 18.3% (*p*=0.02) [108].

In both the IPT and CBASP group [105], HRSD scores decreased after 16 weeks, but only in the CBASP group statistical significance was reached (*t*(13)=3.53, *p*=0.004). BDI scores were significantly lower after 16 weeks in both groups (IPT: *t*(14)=2.34, *p*=0.034; CBASP: *t*(13)=5.01, *p*<0.001). HRSD scores did not show a significant difference between the groups, whereas BDI scores showed a significantly higher reduction in depressive symptoms in the CBASP group after 16 weeks (mean BDI score of 10.79 vs. 21.27 in IPT; *F*(1,26)=4.34, *p*=0.047, treatment effect size: Cohen’s *d*=0.87).

Eight months after the start of the treatments (IPT, nortriptyline, or usual care), all HRSD scores improved significantly (χ^2^=816.14, *df*=6, *p*<0.001), and a significant difference was found between the groups (χ^2^=14.92, *df*=2, *p*=0.001). Post-hoc group *t*-test comparisons showed significant differences (*p*<0.01) in HRSD scores between nortriptyline and usual care, at most measurement times favoring nortriptyline, and between IPT and usual care, favoring IPT after eight months. No significant difference was found between IPT and nortriptyline at any moment in time [106].

### Meta-analysis and summary of findings

**Figure 3:**
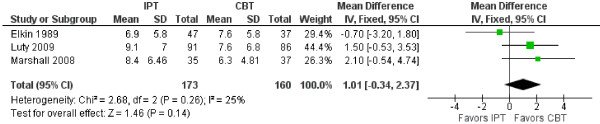
**Comparison of HRSD scores between IPT and CBT. ***CBT* Cognitive Behavior Therapy; *IPT* Interpersonal Psychotherapy; *SD* Standard Deviation.

As can be seen in Table 2, heterogeneity between the studies exists, which made it difficult to make meta-analytic comparisons. However, three studies were comparable in terms of measuring the effects of IPT and CBT [102,103,107]. The mean difference between the treatments was 1.01 (95% CI: -0.34, 2.37) favoring CBT over IPT, but did not reach a statistically significant level. See Figure 3 for more detailed information.

Although no further meta-analyses were possible, and results appeared to be inconsistent, some conclusions can be drawn from these studies. IPT combined with nefazodone improved MADRS scores significantly better than did nefazodone alone [101]. Furthermore, higher HRSD scores were found in IPT patients in comparison with PHT-CM patients [103]. IPT patients showed a significantly greater decrease of HRSD and BDI scores than WLC patients [108]. As measured with the BDI, depressive symptoms were reduced more in CBASP patients in comparison with IPT patients [105]. Finally, IPT patients produced lower HRSD scores in comparison with patients receiving usual care [106].

## Discussion

### Main results

The results of this systematic review show inconsistent findings in the eight heterogeneous studies included. The effectiveness and efficacy of the several treatments is comparable in most studies, and some conclusions may be drawn. Overall, the efficacy of IPT and CBT appears to be equal [102]. Contradictory results were found in IPT in comparison with pharmacotherapy. IPT combined with nefazodone appears to have a higher efficacy than sole nefazodone [101], while pharmacotherapy combined with clinical management appears to have a higher efficacy than IPT alone [103]. However, another study showed comparable results between IPT and imipramine hydrochloride with clinical management (CM), which both returned a better outcome on the HRSD compared to placebo with CM [107]. Furthermore, venlafaxine seems to reduce depressive symptoms more than IPT after six weeks, although this outcome was not significant [104]. The effects of using sole IPT and sole nortriptyline do not significantly differ from each other [106]. IPT and CBASP appear to be very comparable in efficacy, although scores of the BDI showed a slight preference for CBASP [105]. Finally, IPT appears to be more effective than wait list condition [108], and usual care after eight months, as does nortriptyline [106].

These outcomes suggest that several kinds of treatments are effective or efficacious for depressed patients, although one has to keep in mind the small number of included studies. Patients are recommended to choose a treatment which fits their personal preferences, since this may affect the outcome of the treatment. Policy makers are advised to base regulations on the effectiveness and efficacy of treatments in general, instead of a slightly different effect between one treatment and the other, since these studies do not take individual differences and preferences into account.

### Limitations

This review has a number of limitations. First, this review included only adult outpatients with unipolar, non-psychotic major depression as a primary diagnosis. Although these inclusion criteria were a deliberate choice, this review has consequences for the generalizability. These results are not generalizable to children, adolescents, or the elderly, to patients with other kinds of depression, or to patients suffering from a combination of depression and medical conditions, or from depression and substance abuse. Furthermore, no distinction has been made in the severity of depression, which causes a higher heterogeneity in the complete sample, making results more uncertain.

Second, only eight studies with a limited number of participants were included in this review. Although most studies showed a low risk of bias, the small size of the sample may increase this risk. Furthermore, results are harder to generalize with a small number of participants, especially because many different kinds of treatments have been compared with each other (high heterogeneity), which limited the number of participants in the groups not receiving IPT. Moreover, the limited number of included studies in this review, makes one question the applicability of the Cochrane guidelines for conducting a systematic review [52], for clinical treatments in mental health care.

Third, all included patients were outpatients and therefore had to be willing and motivated to participate in the selected studies. This may cause some bias, since not all types of patients could be included in the studies. For example, treatment-resistant depressed patients may have been less motivated than patients who were not treatment-resistant, and it may not be possible to generalize results for these patients.

Fourth, pharmacotherapy consisted of different types of antidepressant medication. Although these medications may seem to be equally effective, some differences may exist, which may interfere with the results of this review. Furthermore, one study [101] used nefazodone as pharmacotherapy, although this medication has been withdrawn in, amongst other countries, the USA and the Netherlands, because of hepatotoxicity associated with this drug [114].

Fifth, some of the findings were based on the scores of the HRSD [101,103-108]. However, this scale has recently been criticized for having multiple problems, including among others the existence of different versions and not being as sensitive as other scales [115,116]. Despite these flaws, the HRSD has been used in many studies and the outcomes of this scale can therefore not be excluded from this review. Furthermore, findings were also based on the MADRS [101,102], which is more sensitive to treatment effect than the HAMD [117], and on the BDI [104,105] which correlates weakly with the HDRS [118] and has several advantages and disadvantages [119], but is widely used.

Sixth, one study [104] measured the efficacy only after six weeks, without follow-up measurement. This is a very short period for measuring the efficacy of IPT. Therefore, the results of this study may be questionable. Furthermore, these authors did not include an intention-to-treat analysis, which increases the risk of bias.

Finally, although a profound search has been performed, there is no complete certainty that all studies eligible for this review have been found. Furthermore, the search was directed only at published studies, automatically excluding unpublished data, causing possible publication bias.

## Conclusions

It can be concluded that the differences between the effects and efficacy of several types of treatment are very small and they are often not significant. This in turn is consistent with a study concluding that the effects of psychotherapy for adult depression in meta-analyses are overestimated [27]. Nevertheless, usual care, as described in the study of Schulberg et al. [106], appears to be ineffective and is not recommended as a treatment for MDD. Therefore, psychotherapeutic treatments such as IPT and CBT, and/or pharmacotherapy are recommended as first-line treatments for depressed adult outpatients. This conclusion is consistent with a previous study [21], and review [26], and previous meta-analyses [28,29,32,33,55],although, as has been stated in the introduction, these studies had several limitations as well. Furthermore, it is recommended that the type of treatment is adjusted to the individual preferences of the patient.

Future research should focus on a larger sample including patients with MDD, while correcting for severity of depression. Since many studies focused on IPT combined with medication, it is recommended that these studies be included in future research as well. Furthermore, it is recommended that future studies included in a review, have longer follow-up periods. All studies should aim for the highest quality standards currently set.

## Competing interests

The authors declare that they have no competing interests.

## Authors’ contributions

MH designed the study with the support of SE and TR. MH undertook the literature search with help from TE, identified potential and final selected articles, interpreted results, drafted and revised all versions of the manuscript, supported by SE and TR. In case of doubt during the screening and analyzing phase, TR was consulted. SE and TR supervised the development of the manuscript. All authors read and approved the final version.

## Pre-publication history

The pre-publication history for this paper can be accessed here:

http://www.biomedcentral.com/1471-244X/13/22/prepub

## Supplementary Material

Additional file 1 Search strategy. Click here for file

Additional file 2 Checklist. Click here for file

Additional file 3 List of excluded studies. Click here for file
