# Supplementary material for: The effectiveness of individual interpersonal psychotherapy as a treatment for major depressive disorder in adult outpatients: a systematic review
Source: BMC Psychiatry. 2013 Jan 11;13:22. doi: 10.1186/1471-244X-13-22 (PMC3558333; doi:10.1186/1471-244X-13-22)
Supplement: Additional file 1 — Search strategy. [file 1471-244X-13-22-S1.doc]

## Additional file 1 – Search strategy

**PubMed 16-08-2012 (via NCBI)**

Concept ‘depression’

1. “Depression”[MeSH]
2. "Depression, Postpartum"[Mesh]
3. "Depressive Disorder, Major"[Mesh]
4. "Dysthymic Disorder"[Mesh]
5. Depress*
6. Dysthymi*
7. 1 OR 2 OR 3 OR 4 OR 5 OR 6

Concept ‘effectiveness’

1. "Evaluation Studies as Topic"[Mesh]
2. "Treatment Outcome"[Mesh]
3. "Program Evaluation"[Mesh]
4. "Comparative Effectiveness Research"[Mesh]
5. "Clinical Trials as Topic"[Mesh]
6. "Clinical Trial" [Publication Type]
7. 8 OR 9 OR 10 OR 11 OR 12 OR 13

Concept ‘IPT’

1. Interpersonal
2. “Psychotherapy”[MeSH]
3. Interpersonal therapy
4. Interpersonal psychotherapy
5. IPT
6. 15 OR 16 OR 17 OR 18 OR 19
7. 7 AND 14 AND 20
8. “Adolescent”[MeSH]
9. “Aged”[MeSH]
10. 22 OR 23
11. (21) NOT 24

*Limits*

English, Dutch, Adult: 19-44 years, Middle Aged: 45-64 years, Publication Date from 1970/01/01 to 2012/08/01

*Search String (with limitations):*

(#21) NOT #24 AND (("1970/01/01"[PDAT] : "2012/08/01"[PDAT]) AND (Dutch[lang] OR English[lang]) AND ("adult"[MeSH Terms:noexp] OR "middle aged"[MeSH Terms]))

Overall results: 2469

**PsycINFO 21-08-2012**

Concept ‘depression’

1. exp Major Depression/
2. exp Postpartum Depression/
3. Dysthymi*.af.
4. 1 or 2 or 3

Concept ‘effectiveness’

1. exp Treatment Effectiveness Evaluation/
2. exp Mental Health Program Evaluation/
3. exp Psychotherapeutic Processes/
4. exp Treatment/
5. exp Treatment Outcomes/
6. exp Psychotherapeutic Outcomes/
7. exp Clinical Trials/
8. 5 or 6 or 7 or 8 or 9 or 10 or 11

Concept ‘IPT’

1. exp Interpersonal Psychotherapy/
2. interpersonal therapy.af.
3. interpersonal psychotherapy.af.
4. IPT.af.
5. 13 or 14 or 15 or 16
6. 4 AND 12 AND 17

*Limits*

Publication Year from: 1970-Current; Age Groups: Adulthood (18 yrs & older), Young Adulthood (18-29 yrs), Thirties (30-39 yrs), Middle Age (40-64 yrs); Languages: English, Dutch

Results after limitations: 993

Overall results after deleting duplicates: 3329
