# Supplementary material for: The effectiveness of individual interpersonal psychotherapy as a treatment for major depressive disorder in adult outpatients: a systematic review
Source: BMC Psychiatry. 2013 Jan 11;13:22. doi: 10.1186/1471-244X-13-22 (PMC3558333; doi:10.1186/1471-244X-13-22)
Supplement: Additional file 2 — Checklist. [file 1471-244X-13-22-S2.doc]

## Additional file 2 – Checklist

**The Delphi List**

1. Was a method of randomization performed?
2. Was the treatment allocation concealed?
3. Were the groups similar at baseline regarding the most important prognostic indicators?
4. Were the eligibility criteria specified?
5. Was the outcome assessor blinded?
6. Were point estimates and measures of variability presented for the primary outcome measures?
7. Did the analysis include an intention-to-treat analysis?

**Risk of bias tool**

1. Random sequence generation (selection bias)
2. Blinding of outcome assessment (detection bias)
3. Incomplete outcome data (attrition bias)
4. Selective reporting (reporting bias)
