# Supplementary material for: The effectiveness of individual interpersonal psychotherapy as a treatment for major depressive disorder in adult outpatients: a systematic review
Source: BMC Psychiatry. 2013 Jan 11;13:22. doi: 10.1186/1471-244X-13-22 (PMC3558333; doi:10.1186/1471-244X-13-22)
Supplement: Additional file 3 — List of excluded studies. [file 1471-244X-13-22-S3.doc]

## Additional file 3 – List of excluded studies

Excluded studies with reason for exclusion

| *Study* | *Reason for exclusion* |
| --- | --- |
| Ablon, et al., 2002 | Based on other study |
| Agosti, et al., 1997 | Based on other study |
| Barber, et al., 1996 | Based on other study |
| Blanco, et al., 2001 | No research data |
| Blom, et al., 2010 | Based on other study |
| Blom, et al., 1996 | No comparison in the study |
| Blom, et al., 2007 | Based on other study |
| Bressi, et al., 2010 | Not IPT as described in eligibility criteria |
| Brown, et al., 1996 | Based on other study |
| Brown, et al., 1999 | Based on other study |
| Browne, et al., 2002 | Wrong diagnosis |
| Bulmash, et al., 2009 | Wrong aim of the study |
| Carter, et al., 2011 | Based on other study |
| Cascalenda, et al., 2002 | Review and/or meta-analysis |
| Coulehan, et al., 1997 | Based on other study |
| Croghan, et al., 1999 | Not IPT as described in eligibility criteria |
| Cuijpers, Dekker, et al., 2009 | Review and/or meta-analysis |
| Cuijpers, et al., 2005 | Not IPT as described in eligibility criteria |
| Cuijpers, et al., 2011 | Review and/or meta-analysis |
| Cuijpers, Van Straten, Bohlmeijer, et al., 2010 | Review and/or meta-analysis |
| Cuijpers, Van Straten, Hollon, et al., 2010 | Review and/or meta-analysis |
| Cuijpers, Van Straten, Schuurmans, et al., 2010 | Review and/or meta-analysis |
| Cuijpers, Van Straten, Van Oppen, et al., 2008 | Review and/or meta-analysis |
| Cuijpers, Van Straten, et al., 2009 | Review and/or meta-analysis |
| Cuijpers, Van Straten, Warmerdam, et al., 2008 | No research data |
| De Maat, et al., 2007 | Review and/or meta-analysis |
| De Mello, et al., 2005 | Review and/or meta-analysis |
| De Mello, et al., 2001 | Not IPT as described in eligibility criteria |
| Dorrepaal, et al., 1998 | Review and/or meta-analysis |
| Dunner, 2001 | No research data |
| Elkin, et al., 1995 | Based on other study |
| Frank, et al., 1990 | Not IPT as described in eligibility criteria |
| Frank, et al., 2000 | Wrong aim of the study |
| Godfrin, et al., 2010 | Not IPT as described in eligibility criteria |
| Guidi, et al., 2011 | Review and/or meta-analysis |
| Hollon, et al., 2005 | Review and/or meta-analysis |
| Hollon, et al., 2010 | Review and/or meta-analysis |
| Imber, et al., 1990 | Based on other study |
| Jakobsen, et al., 2011 | Review and/or meta-analysis |
| Kim, 2003 | Based on other study |
| Kingston, et al., 2007 | Not IPT as described in eligibility criteria |
| Kotova, 2005 | Review and/or meta-analysis |
| Kriston, et al., 2010 | Protocol for a study |
| Kushner, et al., 2009 | Wrong aim of the study |
| Lave, et al., 1998 | Based on other study |
| Markowitz, 1994 | Wrong diagnosis |
| Markowitz, 1996 | Wrong diagnosis |
| Markowitz, et al., 2005 | Wrong diagnosis |
| Miller, et al., 1996 | No research data |
| Pampallona, et al., 2004 | Review and/or meta-analysis |
| Reinceke, et al., 2000 | No research data |
| Reynolds, et al., 1996 | Based on other study |
| Schene, et al., 2007 | Not IPT as described in eligibility criteria |
| Schulberg, et al., 1997 | Not IPT as described in eligibility criteria |
| Schulberg, et al., 1995 | Wrong aim of the study |
| Segal, et al., 2001 | Based on other study |
| Shea, et al., 1992 | Based on other study |
| Svanborg, et al., 2008 | Wrong diagnosis |
| Thase, et al., 1997 | Review and/or meta-analysis |
| Van Roijen, et al., 2006 | Not IPT as described in eligibility criteria |
| Watkins, et al., 2003 | Based on other study |
| Weissman, 2007 | Review and/or meta-analysis |
